# Synthesis and Characterisation of a Graphene Oxide-Gold Nanohybrid for Use as Test Material

**DOI:** 10.3390/nano13010033

**Published:** 2022-12-21

**Authors:** Taiwo Hassan Akere, Aline M. Z. de Medeiros, Diego Stéfani T. Martinez, Bashiru Ibrahim, Hanene Ali-Boucetta, Eugenia Valsami-Jones

**Affiliations:** 1 School of Geography, Earth and Environmental Science, College of Life and Environmental Sciences, University of Birmingham, Birmingham B15 2TT, UK; 2 Nanomedicine, Drug Delivery & Nanotoxicology (NDDN) Laboratory, School of Pharmacy, College of Medical and Dental Sciences, University of Birmingham, Birmingham B15 2TT, UK; 3 Brazilian Nanotechnology National Laboratory (LNNano), Brazilian Centre for Research in Energy and Materials (CNPEM), Campinas 13083-100, SP, Brazil; 4 Centre of Nuclear Energy in Agriculture (CENA), University of São Paulo (USP), Piracicaba 13416-000, SP, Brazil

**Keywords:** graphene oxide, gold, nanohybrid, nanoparticles, ecotoxicology, nanotoxicity

## Abstract

This paper reports the synthesis and characterization of a graphene oxide–gold nanohybrid (GO-Au) and evaluates its suitability as a test material, e.g., in nano(eco)toxicological studies. In this study, we synthesised graphene oxide (GO) and used it as a substrate for the growth of nano-Au decorations, via the chemical reduction of gold (III) using sodium citrate. The GO-Au nanohybrid synthesis was successful, producing AuNPs (~17.09 ± 4.6 nm) that were homogenously distributed on the GO sheets. They exhibited reproducible characteristics when characterised using UV-Vis, TGA, TEM, FTIR, AFM, XPS and Raman spectroscopy. The nanohybrid also showed good stability in different environmental media and its physicochemical characteristics did not deteriorate over a period of months. The amount of Au in each of the GO-Au nanohybrid samples was highly comparable, suggesting a potential for use as chemical label. The outcome of this research represents a crucial step forward in the development of a standard protocol for the synthesis of GO-Au nanohybrids. It also paves the way towards a better understanding of the nanotoxicity of GO-Au nanohybrid in biological and environmental systems.

## 1. Introduction

Since the advent of nanotechnology in the 1980s, our society has been transforming remarkably, and will likely continue to do so to meet our growing and evolving needs. Faster and powerful computers, more accurate medical devices, sunscreens, cost- and quality-efficient water filters are some of the remarkable applications involving nanotechnology. From environmental pollution, to increasing energy demand, scarcity of potable water and biomedical constraints, the quest for solutions to some of the world’s problems has driven intense research into nanomaterials [1]. On the forefront of these applications are carbon-based nanomaterials, such as graphene and its derivatives [graphene oxide (GO), reduced graphene oxide (rGO)], carbon nanotubes [single and multi-walled nanotubes] and carbon dots [2].

Graphene-based materials represent a recent addition to the nanomaterials family and yet have already found use in many applications, for example, in electronics, medicine, agriculture and energy, and have been incorporated in many consumer products: drug nanocarriers, high-capacity batteries, lightweight and strong materials for automotive and aerospace, biosensors, biomedical devices, water desalination, anti-corrosion coatings, etc. [3,4,5,6].

More recently, research work has focused on exploring multicomponent nanohybrids, which offer the advantage of combining multiple functionalities into a single advanced material [7,8,9]. Popular combinations of such hybrid materials are those involving metal or metal oxide nanoparticles (like TiO_2_, ZnO, Au, CuO) and carbon nanomaterials (such as graphene oxide, carbon nanotubes, fullerenes). For instance, both GO and silver nanoparticles are known to exhibit antibacterial properties [10,11]. However, graphene oxide–silver nanocomposites (GO-Ag) have been reported to have a superior antimicrobial capability compared to their individual components [12,13].

Similarly, graphene oxide–gold nanohybrids (GO-Au) have been reported to demonstrate synergistic features beyond their individual capabilities, such as increased electrical conductivity, catalytic activity, higher surface area, optical and medical properties, etc. [14,15]. These features have rapidly increased the use of this nanohybrid in various applications, such as cancer detection, bioimaging and biosensing [16,17,18].

Unlike natural nanomaterials that plants and animals have adapted to, engineered materials (especially emerging ones such as nanohybrids) pose novel threats that require critical evaluation of their potential harm to humans and ecosystems. As with any new discovery, there are concerns about the potential of novel nanohybrids to cause toxicity over and above that of their individual components [19,20]. Their safety to the ecosystem and human health is yet to be fully demonstrated since the very features that make them so advanced may also be the source of concern to ecotoxicologists, specifically in terms of their ability to bypass biological barriers. A number of studies have demonstrated adverse effects to human cells [21], bacteria [22], algae [23], water fleas [24], fish [25], rodents [26] and marine mammals [22].

Currently, there is no standardised protocol for the synthesis of GO-Au nanohybrids. It is, therefore, unclear how, in different formulations, the two components are conjugated, quantitatively and qualitatively, and whether performance, but also potential toxicity, may be affected. Most of the available research work on GO-Au has focused on the applications of the nanohybrid [16,17,27]. For assessing its safety, however, it is important that critical physicochemical parameters of the GO-Au nanohybrid are evaluated and correlated to its toxicity. This is to ensure the reproducibility and compatibility of the results.

Herein, the production of a graphene oxide–gold nanohybrid that will be suitable as a test material was carefully designed and evaluated. Specifically, the aspects that were researched include: (i) synthesis and characterization of GO and GO-Au, (ii) stability of the nanohybrid in different environmental media (iii), quantification of Au NPs in the GO-Au nanohybrid and (iv) evaluation of ageing effects on the nanohybrid.

## 2. Materials and Methods

### 2.1. Materials

Graphite flakes, sodium nitrate (NaNO_3_), sulphuric acid (H_2_SO_4_, 98% *w*/*w*), potassium permanganate (KMNO_4_), hydrogen peroxide (H_2_O_2_, 30% *w*/*w*), chloroauric acid (HAuCl_4_), tannic acid (C_76_H_52_O_46_) and trisodium citrate (Na_3_C_6_H_5_O_7_) were all purchased from Sigma Aldrich (Dorset, UK). Each of the chemicals were used as purchased without additional purification. Ultrapure water was used in all the experiments.

### 2.2. Synthesis of Graphene Oxide (GO)

GO was synthesised using a modification of the Hummer’s method [28]. In brief, 5.0 g of graphite flakes, 3.75 g of sodium nitrate (NaNO_3_) and 370 mL sulphuric acid were mixed in a 1 L twin neck round bottom flask under magnetic stirring at ambient temperature. After 10 min, the mixture was cooled in an ice bath for another 10 min. 22.5 g of potassium permanganate was added slowly to the mixture while stirring until it changed to a dark green paste. The stirring continued for 72 h in a fume hood at room temperature. Then, the mixture was diluted with 500 mL of ultrapure water and stirred continuously for 1 h at 95 °C. A rapid temperature increase and violent effervescence was observed. After the temperature was reduced to ~60 °C, 15 mL of hydrogen peroxide (3%) was added drop-wise to the mixture to reduce the potassium permanganate. The mixture was stirred continuously overnight at room temperature. Following that, a yellow tint coloration was observed.

The suspension was then transferred into two 50 mL tubes for ease of handling and vigorously mixed with a vortex. The solution was centrifuged twice at 7000 rpm for 15 min at 25 °C. The precipitate was then treated with 30 mL sulphuric acid, 5 mL hydrogen peroxide and 965 mL ultrapure water to remove organic impurities and oxidant ions. The resultant mixture was stirred with a glass rod and centrifuged again (7000 rpm). The black residue was re-suspended with water and passed through dialysis tubing (cut-off: 14,000 kDa) in ultrapure water for 72 h. The purified GO (45 mL) was freeze-dried (Berta 1–8 LSCplus, Christ, Herlev, Denmark), stored in a sealed bottle and kept in a desiccator until further experiments were performed.

### 2.3. Synthesis of Graphene Oxide-Gold Nanohybrid (GO-Au)

The GO-Au nanohybrid was synthesised using a modification of the method developed by Song et al. (2014). In brief, 60 mg of the synthesised GO was dispersed in 750 mL of ultrapure water at room temperature under magnetic stirring for 1 h. Then, chloroauric acid (150 mg) was added to the GO dispersion. The mixture was then stirred continuously for 1 h, after which, a solution of sodium citrate (75 mL) was added and stirred for a further 30 min. The mixture was then heated to 80 °C for 1 h. The final dispersion was then diluted with ultrapure water and centrifuged 3 times (at 7000 rpm for 15 min each time). The supernatant was discarded and the final GO-Au nanohybrid was collected. The nanohybrid was purified using dialysis tubing (cut-off: 14,000 kDa) for 48 h. Purified GO-Au (82 mL) was stored in a refrigerator at 4 °C.

### 2.4. Characterization of the Nanohybrid and Its Components

The UV-vis absorbance and spectra of GO and GO-Au nanohybrid were measured by UV-2600 Shimadzu spectrophotometer using quartz cells. The spectra for the samples were measured from 800–200 nm. The surface charge (zeta potential) of the GO dispersion was measured using Malvern Zetasizer. 1 mL of the sample was placed in a zeta cell and measured at room temperature. Mean Z value was obtained for three replicate measurements. The Raman spectrum was obtained using Renishaw InVia. The samples were sandwiched between two glass slides to reduce particle size. The green laser (532 nm edge), which has illumination range suitable for inorganic materials, was used for the measurement.

To obtain TEM image, a single drop of the homogenised sample was deposited on a Formvar/carbon grid. The deposit was left to dry off and then examined under a Joel 1400 Bio TEM using an 80 KV electron beam current. An AFM scanning was done by depositing drops of samples on a glass slide and left to dry off. The slide was then mounted on the microscope. Imaging of the samples was done in peak force tapping using multimode 8 microscope with Nanoscope 5 controller (Brucker, Durham, UK). Fourier-transform infrared spectroscopy (FTIR) analyses was done using FT-IR spectrometer Frontier MIR (Perkin Elmer, Beaconsfield, UK). Thermogravimetric (TGA) was performed using Perkin Elmer’s TGA 8000 (10 °C/min to 1000 °C; synthetic air flow rate of 50 mL/min).

X-ray photoelectron spectroscopic (XPS) analysis was carried out to investigate the internal and surface chemistry of GO and GO-Au nanohybrid using Thermo Scientific K-Alpha XPS. To identify the functional groups and chemical state of gold, spectra of high resolution for carbon and gold (nanohybrid) were acquired and analysed with Thermo Avantage software (version 5.957, ThermoFisher, Basingstoke, UK).

### 2.5. Quantification of Au NPs in the GO-Au Nanohybrid

The concentration of Au nanoparticles in the nanohybrid was quantified by inductively coupled plasma–mass spectrometry (ICP-MS) (Perkin Elmer NexION 300X). 100 µL of the GO-Au nanohybrid were digested in 900 µL of HCl (concentration of 15M) overnight. An aliquot of the digested sample was further diluted using 2% HCl to ppb levels. Afterwards, the sample was centrifuged at 7500 rpm for 15 min. The supernatant was characterised with UV-vis spectrophotometer for the detection of GO. In the absence of GO, the supernatant was analysed for AuNP on ICP-MS. Five replications of this measurement were performed.

### 2.6. Dispersion Stability

The dispersion stability of the GO and GO-Au (both at 100 mg L^−1^) in ultrapure water, borehole water and high hardness combo media (see Supplementary Materials for quality parameters), with and without natural organic matter (NOM) (Suwannee River NOM; International Humic Substances Society; final NOM concentration: 20 mgL^−1^) was monitored using a modification of the OECD (Organisation for Economic Co-operation and Development) 318 guideline. The samples were kept static, and an aliquot (100 µL) was taken from the surface of the dispersion at 0, 24 and 48 h. Care was taken during aliquot sampling to avoid agitating the whole sample. The stabilities of the nanomaterials were compared in triplicate using the detection of GO at an absorbance of 230 nm, measured using spectrophotometric analyses (microplate spectrophotometer, Spark, Tecan, Reading, UK)). The chemical constituents of the NOM are [% *w*/*w* of dry, ash-free sample]: water (5.69), ash (4.01), carbon (50.7), hydrogen (3.97), oxygen (41.48), nitrogen (1.27) and sulphur (1.78).

## 3. Results and Discussion

### 3.1. Characterization of Graphene Oxide

**Figure 1:**
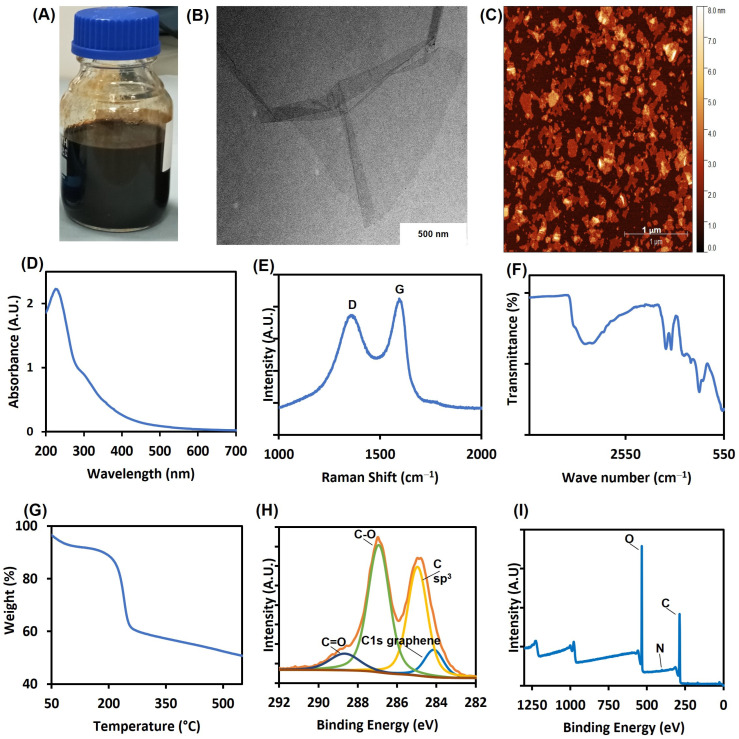
Characterization of graphene oxide (GO) prepared using the modified Hummer’s method. (**A**) Purified GO stock dispersion a concentration of 2 mg/mL in ultrapure water, (**B**) TEM image of GO dispersion, (**C**) AFM topographic image (**D**) UV-vis absorption spectrum, (**E**) Raman spectrum, (**F**) FTIR pattern, (**G**) thermogravimetric analysis spectrum (**H**) high resolution C1s XPS spectrum, (**I**) survey XPS spectrum.

As can be seen in Figure 1A, the final yield post-synthesis of GO exhibited a dark brown colour, which is characteristic of GO. An aliquot of the GO was freeze-dried for further characterization and to determine its concentration (1.0 g/L). The morphology of the GO was evaluated using transmission electron microscopy (TEM) and atomic force microscopy (AFM) (Figure 1B,C). Using the TEM method described by Kumar et al. [29] for estimating the number of layers, the GO sheets were mostly single layer and exhibited flake-like structure, as shown in Figure 1B. This is corroborated by the cross-sectional height profile of the GO flakes thickness measured by AFM height scan (Supplementary Materials, Figure S1). The measurement showed 1 nm thickness for most of the GO flakes. According to recommended classification [2,5], the GO sheets fall within single to few layer GO.

UV-vis spectroscopy was performed to examine the extent of oxidation of the GO sheets. The absorption spectra can be used as a mechanism of identifying GO sheets. As shown in Figure 1D, the UV-vis spectrum of the GO sample shows a sharp maximum peak around 230 nm which indicates π→π* transitions of C=C bonds. The spectrum also showed a weaker peak (shoulder) at around 300 nm which indicates n→π* transitions of C=O bonds. This result confirms that the prepared sample contains GO, and is consistent with the UV-vis spectra described by others [30,31,32].

As expected, the mean zeta potential values of the GO samples was −56.4 ± 2.4 mV (pH = 7.9), suggesting a negative charge and a good stability [33]. The influence of pH on zeta potential (measure of surface charge) can have implications in environmental fate. The weak forces that bind nanoparticles together when they agglomerate can be altered by changes in pH [34]. In toxicity exposures where pH is expected to be maintained within certain range, a moderately stable suspension of particles provides some confidence on a low likelihood of agglomeration occurring in low ionic potential media.

In addition, Raman spectroscopy, a non-destructive method, was used to obtain structural information on the carbon-based nanomaterials as suggested by others [35]. It is used to determine the stacking order, defects and number of layers of graphene [36,37]. GO normally has two peaks: the D peak falls within the 1300–1400 cm^−1^ range, while the G peak falls within the 1500–1600 cm^−1^ range. The D band indicates a defect region and the G band indicates a graphitic region [31]. For the GO described here, as shown in Figure 1E, the D peak was observed at 1343 cm^−1^ and the G peak appeared at 1586 cm^−1^. This further confirms the presence of GO in the synthesised sample. Additionally, the I_D_/I_G_ ratio of 0.84 was recorded, indicating high level of defects, showing that this GO has undergone an oxidation process and intense exfoliation.

To identify the various surface functionalities on the GO, The FTIR technique was employed. The FTIR spectrum for our GO sample is shown in Figure 1F. The graphite oxidation resulted in the formation of the following broad bands: 3212 cm^−1^ matching the O-H stretching vibrations that are typical of hydroxyl and carboxyl functional groups [32,38,39]; 1719 cm^−1^ corresponding to C=O stretching vibrations implying the presence of carbonyl and carboxyl groups [40]; 1622 cm^−1^ showing the contribution of cyclic aromatic groups; 1050 cm^−1^ matching the C-O stretching vibrations, which are the typical absorption bands of ethers [37,40].

In order to assess the thermal stability and behaviour of the synthesised GO, TGA analysis was performed, as shown in Figure 1G. The GO exhibited two decomposition stages at approximately 190 °C and 550 °C, respectively. The initial weight loss up until 100 °C resulted from the evaporation of water content, while the loss from 150–200 °C can be attributed to the loss of hydroxyl and acidic functional groups [41]. The final weight loss up to 700 °C originated from the breakdown of carbonyl groups formed during the process of GO oxidation [42].

The chemical make-up of the GO surface was evaluated by XPS and the results are presented in Figure 1H,I. The XPS spectra represents the intensity of photoelectrons emitted from the various atoms on the GO surface. The analysis of the XPS survey spectrum showed that the surface chemistry of the GO is composed of 68.15% carbon and 31.85% oxygen. The elevated percentage of oxygen confirms the oxidation of GO. The absence of commonly reported impurities such as sulphur and nitrogen indicate the high level of purity of the GO dispersion. The C1s scan shows the different carbon-oxygen groups present on the GO surface (Table 1).

### 3.2. Characterization of Graphene Oxide-Gold Nanohybrid (GO-Au)

**Figure 2:**
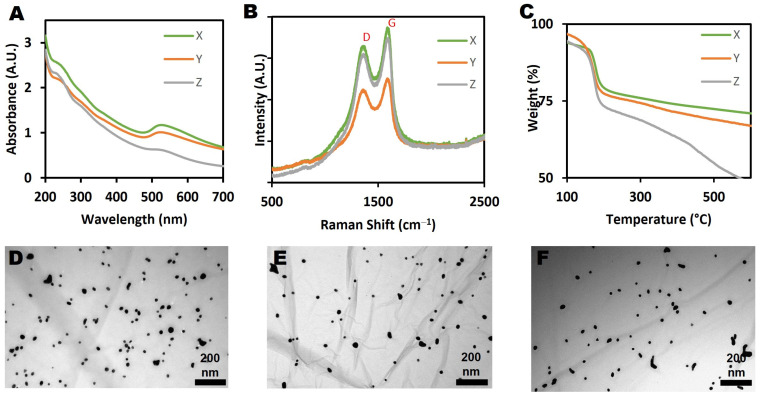
Characterization of three batches of GO-Au nanohybrid (X, Y & Z): (**A**) UV-vis absorption spectrum, (**B**) Raman spectrum, (**C**) thermogravimetric analysis spectrum, (**D**–**F**) TEM images corresponding to each of the 3 replicate nanohybrids.

Three different batches of the GO-Au nanohybrid (X, Y and Z) were prepared and compared, as shown in Figure 2. The synthesis method was optimised to produce the highest yield of GO-Au nanohybrid possible, over 80 mg (>2 mg/mL). Previous studies have produced GO-Au nanohybrid suspensions within the range of 5–20 mL [43,44,45,46]. On characterization, it was found that all the three batches were very similar, suggesting reproducibility of the modified synthesis method of the GO-Au nanohybrid. It is important to note that there are major differences between this modified synthesis protocol and the original protocol of Song et. al. [44]. Firstly, the original protocol has been scaled-up significantly. In the work presented here, 60 mg of graphene oxide and 150 mg of chloroauric acid were used as against the 4 mg of graphene oxide and 5–20 mg of chloroauric acid of the original protocol. Scaling up or down in the synthesis of nanoparticles can induce significant chemical changes on the final product [47], and it may not result in a product with properties corresponding to those of the original synthesis. Furthermore, the modified synthesis conditions may result in a product of variable consistency, particularly in the case of a hybrid material.

Additionally, the optimised protocol presented here produced a significantly larger yield, which cannot always be guaranteed when scaling up, but which was an essential requirement for a material that would be recommended for toxicological studies, where significant quantities are necessary to allow for testing a range of concentrations and carrying out replicate tests. The final yield of the GO-Au nanohybrid from our work was over 80 mg. Although the yield from the original protocol was not reported, judging by the volume of the reagents used, it is likely to have been less than 4 mg. It is generally recognised that scaling up of nanoparticle synthesis does not automatically translate to high yield of good quality products [48] and addressing this limitation was central to our study.

Lastly, a crucial element in the optimised protocol is the introduction of purification at the end of the synthesis to remove impurities, such as by-products generated during the synthesis process. These impurities may influence the behaviour, bioavailability and toxicity of a nanomaterial [49]. Since the focus of our synthesis is for (eco)toxicological studies, it is important to rid the GO-Au nanohybrid of any impurities. The original protocol, however, was designed for a different application that did not require purification.

The UV-vis spectra of the nanohybrid (Figure 2A) indicated the presence of AuNPs on the GO sheets. Typically, the surface plasmon resonance for AuNPs is seen as a peak at around 520–570 nm. As shown in Figure 2B, the integrity of the graphene sheets was not affected by the AuNPs conjugation, as made evident by the presence of D and G peaks in the Raman spectra of the nanohybrid. Notably, there was a small increase of the I_D_/I_G_ ratio calculated for the GO-Au nanohybrid from 0.84 to 0.89 following the functionalization of Au NPs onto the GO surface. This suggests that defects were introduced onto the GO surface as a result of the attachment of the AuNPs. This is very similar to what Song et al. [44] reported when pure GO sheets were conjugated with Au NPs and showed that the I_D_/I_G_ ratio increased from 0.77 to 0.82 after conjugation. The thermal stability analyses of the GO-Au nanohybrid revealed that the three samples behaved similarly, as shown in Figure 2C. Approximately 20% of original weight was lost at around 200 °C, which corresponds to the evaporation of moisture and hydroxyl or acidic moieties, while another 10–20% of weight was lost around 400 °C, which may be attributed to the decomposition of carbonyl functional groups [50]. The thermal stability of the nanohybrids thus reflects the gradual decomposition of its GO component, and correlates well with the TGA results of the pure GO described earlier, with small deviations likely due to the presence of AuNPs.

As shown in the TEM images of the nanohybrids (Figure 2D–F), both GO and AuNPs can be visually observed on the images, confirming the functionalization of AuNPs onto the surface of the GO sheets. In addition, the average size of the AuNPs was found to be around 17.09 ± 4.6 nm from the TEM analysis. The even and planar shape of the GO sheets provided a suitably high surface area for the attachment of the AuNPs. Bonding of AuNPs to GO surfaces occurs via noncovalent bonds such as hydrogen bonds and electrostatic interactions [51]. As can be seen in Figure 2D–F, AuNPs show limited tendency to aggregate in all the nanohybrid batches. This is not unexpected, as the likelihood of aggregation increases as the quantity of the starting HAuCl_4_ is increased [44].

XPS was employed to determine the chemical composition of the GO-Au nanohybrid. The C1s scan and survey spectrum results are presented in Figures S3 and S4. The GO-Au nanohybrid showed 67.15% of carbon, 29.83% of oxygen and 3.02% of gold (Table 2). Similar to the GO alone, the high oxygen content in the GO-Au nanohybrid is due to oxidation, and the absence of any further features in the spectra confirms purity of the nanohybrid. Deconvolution of the high resolution C1s spectrum, as highlighted in Table 1, revealed the percentage content of the respective functional groups on the GO-Au nanohybrid.

In addition, FTIR analysis of the GO-Au nanohybrid showed to be similar to that of GO alone, as shown in Figure S2. The observed signals between 3200–3700 cm^−1^ match those of hydroxyl groups and carboxyl acids while the absorptions at 1720 cm^−1^ are due to carbonyl groups. The cyclic aromatic groups and the ethers can be identified by the signals at 1622 and 1030 cm^−1^, respectively.

### 3.3. Evaluation of Nanohybrid Ageing

**Figure 3:**
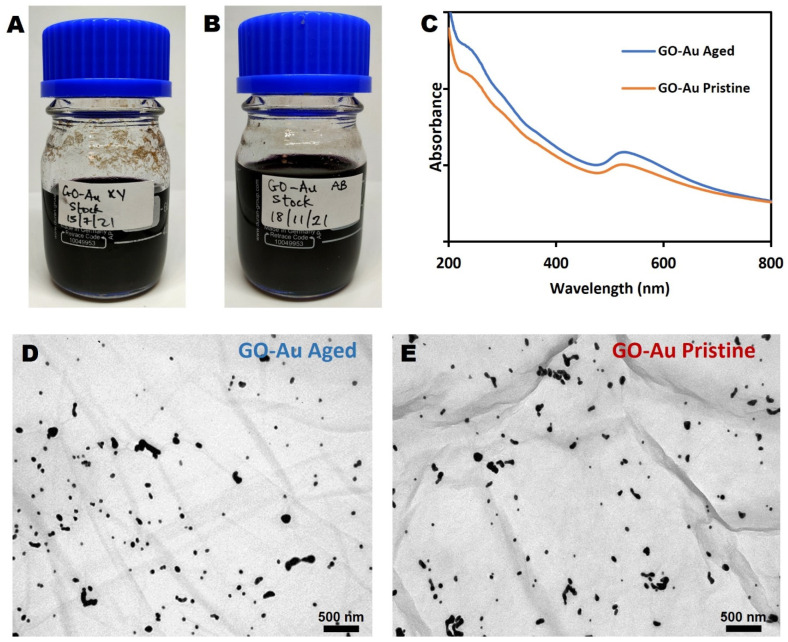
Characterization of pristine and aged (by 4 months) GO-Au nanohybrid. (**A**) Stock of aged nanohybrid (GO-AU XY), (**B**) Stock of pristine nanohybrid (GO-AU XY) (**C**) UV-Vis spectra of the two nanohybrids, (**D**) TEM image of GO-AU Aged (**E**) TEM image of GO-AU Pristine.

The effect of ageing on the quality of the GO-Au nanohybrid was assessed using two nanohybrids produced 4 months apart. Aged GO-Au was produced in July 2021, while pristine GO-Au was produced in November 2021 (Figure 3A,B). Both nanohybrids were stored for a month in darkness at a temperature of 4 °C.

Figure 3C presents the UV-Vis of the pristine and aged nanohybrids, highlighting that both nanohybrids show a maximum peak at around 230 nm, which is characteristic of GO. In addition, both nanohybrids include AuNPs, as indicated by the plasmon band seen around 520 nm, which is distinctive of AuNPs. Apart from the slight difference in absorbance, which is due to the difference in sample concentration, the two nanohybrids appear identical. This suggests that there has been no significant effect of ageing on the samples.

In order to investigate any visual or morphological changes between the pristine and the aged nanohybrids, samples were examined under TEM. As shown in Figure 3D,E, the TEM images confirm that the successful conjugation of the AuNPs was not affected by ageing as well as the presence of single layered GO. In addition, the size distribution of the AuNP is still similar in both aged and pristine nanohybrids.

The stability of the synthesised GO-Au nanohybrid could be due to the mild conditions of the synthesis, which involved only moderate temperature (80 °C). This indicates that the sp2 hybridization of graphene-based materials is stable at moderate temperatures (≤120 °C), which is in agreement with other literature [52,53]. Graphene oxide, however, becomes generally unstable at high temperatures, above 200 °C [54].

The toxicity and reactivity of nanomaterials can be sensitive to ageing and chemical transformations. Depending on the chemical constituent of the media, ageing can influence nanomaterials both structurally and compositionally and, in turn, their interaction with other molecules in the media as well as bio-uptake. The lack of evidence of ageing in the GO-Au nanohybrid adds to the weight of evidence that the proposed synthesis protocol produces a nanohybrid that is suitable for toxicity testing and will remain stable even for longer term exposures, which are not uncommon in ecotoxicity testing.

### 3.4. Quantification of AuNP on GO-Au Nanohybrid

The use of ICP-MS has benefits beyond just characterization of nanoparticles; it is a useful technique for the quantification and detection of nanoparticles in biological assays [55]. Specifically, the accuracy of using ICP-MS to determine AuNP has been well established [56,57,58]. Investigating quantitatively the presence of AuNP in a sample is crucial to understanding their environmental and biological effects [56,59].

Gold standard solutions were used to generate a range of gold concentrations (0.01–1.0 mg/L) for calibrating the ICP-MS instrument.

The concentration of Au in each of the three batches of the GO-Au nanohybrid was measured using the ICP-MS. The average concentration of Au as measured in each of the X, Y and Z replicates was 0.128 ± 0.005, 0.144 ± 0.003 and 0.128 ± 0.01 mg/L, respectively, which corresponds to an average of 1300 mg/L of Au in the neat suspension of the GO-Au nanohybrid (Table S3).

The similarity in the results demonstrates the reproducibility and reliability of our optimised protocol for the synthesis of the GO-Au nanohybrid. Knowing the concentration of AuNP will allow a better understanding of the toxicity of GO-Au nanohybrids in environmental media [60]. In addition, AuNPs can be used as chemical labels to determine the fate and transport of GO-Au in biological or environmental systems [61]. This is particularly relevant for GO, as its compositional tracking against the carbon dominated background of environmental matrices is especially challenging. Instead, GO can be traced through this compositionally stable nanohybrid, involving measurement of Au concentration by, for example, ICP-MS, as a proxy for the presence of the GO-Au nanohybrid.

### 3.5. Assessment of the Impact of NOM on Dispersion Stability of the GO-Au

Evaluation of the stability of nanomaterial dispersions is one of the properties that have been identified as fundamental in understanding their transport and fate in the environment [55,59], as it may influence their cellular and environmental uptake and transportation. In addition, the stability of nanomaterial dispersions is crucial to their holistic ecotoxicological assessment [62]. It is, therefore, essential that this evaluation is done in media that truly represent the likely environmental conditions for the ecotoxicity indicator species under investigation.

**Figure 4:**
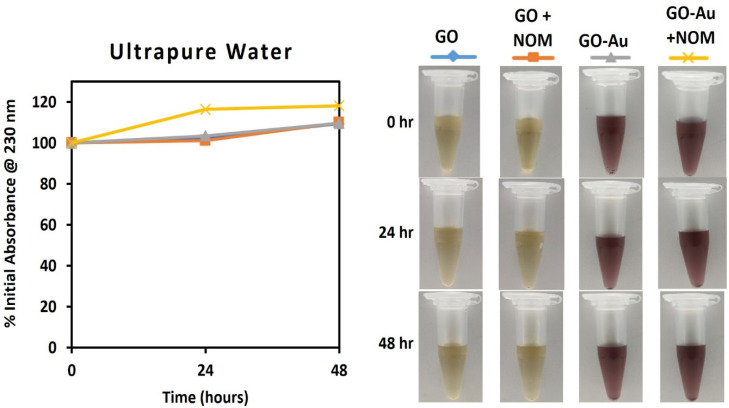
Monitoring of dispersion stability of GO and GO-Au in ultrapure water with and without NOM (20 mg/L). (**left**): Absorbance measured in UV-vis; (**right**): Photographs for visual observation.

**Figure 5:**
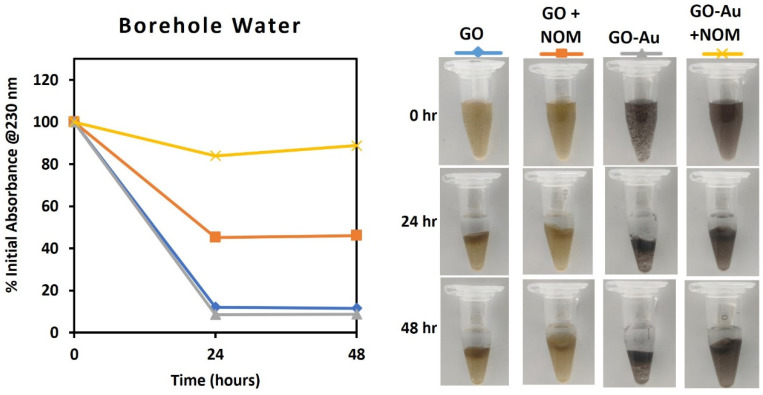
Monitoring of dispersion stability of GO and GO-Au in borehole water with and without NOM (20 mg/L). (**left**): Absorbance measured in UV-vis; (**right**): Photographs for visual observation.

**Figure 6:**
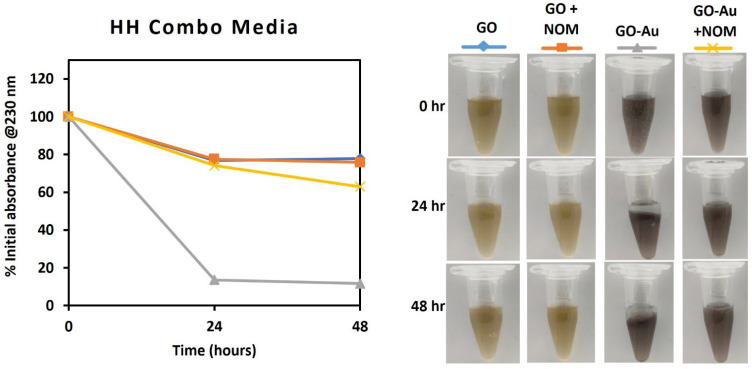
Monitoring of dispersion stability of GO and GO-Au in HH Combo media with and without NOM (20 mg/L). (**left**): Absorbance measured in UV-vis; (**right**): Photographs for visual observation.

Taking a further step towards demonstrating the relevance of the studied nanohybrid as a model material in ecotoxicity research, the stability of GO and GO-Au was evaluated in three media: ultrapure water (control), a natural borehole water (BHW) and a synthetic high hardness combo (HHC) medium (Figure 4, Figure 5 and Figure 6). While BHW is widely used in ecotoxicological studies for culturing many freshwater and marine organisms such as fish, daphnia, clams, etc., HHC as an artificial medium has also been approved for use in culturing and testing model organisms in order to reduce variability in ecotoxicological testing results [63]. The use of both natural (BHW) and synthetic (HHC) media for toxicity testing is recommended by the US EPA, OECD and ISO [64,65,66].

The stability of GO and GO-Au in the studied media will be influenced by environmental variables such as natural organic matter (NOM), pH and the concentrations of cations and anions [32]. NOM tends to bind with particulate matter through hydrogen bonds, steric repulsive forces and Lewis acid-base interactions, thereby increasing the stability and subsequent bioavailability of suspended particles to aquatic organisms [67]. 

In ultrapure water, both the GO and GO-Au nanohybrid demonstrated high stability for 48 h, as shown by the absence of reduction in absorbance at 230 nm (Figure 4). This could be explained by the elevated surface charge of the nanomaterials (ζ_GO_ = −56.4 ± 2.4 mV and ζ_GO-Au_ = −47.4 ± 2.5 mV) as the dispersion stability of suspended particles depends mainly on the repulsive forces due to surface charge [68,69].

However, in the borehole and HH Combo media, the absorbance of GO and GO-Au dispersion after 48 h declined to less than 20% of the initial absorbance, except for the GO in the HH Combo media, which decreased to nearly 80%, as shown in Figure 5 and Figure 6. The surface charges measurements also significantly decreased to ζ_GO_ = −23.6 ± 0.9 mV and ζ_GO-Au_ = −18.3 ± 0.6 mV for borehole, and ζ_GO_ = −34.2 ± 0.8 mV and ζ_GO-Au_ = −22.4 ± 1.4 mV for HH Combo. The decrease of the dispersion stability could be because of the increase of ionic strength of the medium and the adsorption of protons and cations (such as Mg^2+^, Na^+^) from the media onto the GO and GO-Au surfaces [70]. This facilitated aggregation and flocculation of the suspended particles that could also be visually observed (Figure 5 and Figure 6).

The colloidal stability of the two GO materials in the presence of NOM was observed to be better than in its absence. After 24 h in borehole media, the GO and GO-Au nanohybrid maintained approximately 50% and 80% of their initial absorbance respectively. The values increased to 60–80% in the HH Combo media for the same duration. The concentration of the NOM at 20 mg/mL as recommended by OECD was sufficient to make the GO-Au nanohybrid stable in the test media. This emphasises the role of NOM in stabilising suspensions in different environmental media, which was previously reported for other carbon-based nanomaterials [71,72,73]. It could therefore be suggested that the GO-Au nanohybrid can become bioavailable to aquatic organisms in any ecotoxicological study with the tested media.

Media play a crucial role in the fate, behaviour and toxicity testing of nanomaterials. As such, standard test media that contain NOM are preferred for toxicological assessments, as they represent more realistic environmental conditions. The good stability performance of the GO and GO-Au nanohybrid in NOM-containing borehole and HH combo underscores the active role of NOM in bio-nano interaction. These results further demonstrate that the proposed synthesis method can produce GO-Au nanohybrids that are stable in relevant media and may be bioavailable to model organisms in a natural environment. This is an important requirement for ecotoxicological research because biological responses are only triggered when exposure and/or uptake occurs between the GO-Au nanohybrid and the biological target.

### 3.6. Overall Assessment of the GO-Au Nanohybrid as a Model Nano(eco)toxicity Material

The need to develop well-characterised standardised nanomaterials as a means towards improving reproducibility in nano(eco)toxicity studies is well established e.g., [74], and yet, the availability of such standards is limited. Notwithstanding efforts from international standardisation committees, such as OECD and ISO, and a limited number of standard nanomaterials available by the US NIST (National Institute of Standards and Technology), Europe’s JRC (Joint Research Centre) and a few other national standards institute, activity around standardised nanomaterials has remained limited despite early calls for their need [75]. Work towards standards development is often perceived as applied research that may not generate publishable outputs. Additionally, there is confusion about what constitutes a standard material, with terms such as “test nanomaterial”, “reference nanomaterial” and “certified reference nanomaterial” all referring to increasing levels of scrutiny in their production and characterisation [75]. As a result, nanomaterials standards are limited, and when available, they are expensive to acquire and usually available only in very small quantities. This is a concern for toxicology, but even more so for ecotoxicology, where experimental designs (often involving in vivo trials) require large quantities of material.

To our knowledge, there have been no previous attempts to initiate a standardisation process for anything but the simplest and most commonly used nanomaterials, and thus, the nanohybrid presented here is a novel step in the standardisation direction. The data presented above establish the nanohybrid’s reproducibility, provide a high level of characterisation information, notably in ecotoxicity relevant matrices, and demonstrate the potential to produce quantities suitable for large-scale experimentation. In addition, our results show that ageing up to 4 months does not affect the physicochemical properties of the hybrid. Previous work on aged nanomaterials has demonstrated clear differences in toxicological response, when ageing affects material properties e.g., [76], and thus, it is important that stability for a defined period of time has been established. Ecotoxicological studies, particularly when environmentally relevant concentrations of the test material are being assessed, may require longer term assessment, and thus, it is important to have confidence that the test material will not age during testing.

Despite the progress achieved here, further work would be required for the nanohybrid to go beyond a simple test material and to become a reference material. Future work should, for example, demonstrate that the nanohybrid is clear of endotoxin, particularly for toxicity experimentation, and that it can be synthesised in other labs following the protocol presented here and maintain the same physicochemical properties.

## 4. Conclusions

In this study, we have synthesised a GO-Au nanohybrid and optimised the ratio between GO and AuNPs in the nanohybrid produced. The nanohybrid was found to have uniform distribution of AuNPs on the GO sheets and exhibited good stability. This is the first study to show a reproducible standardised protocol for the synthesis of GO-Au nanohybrids. Standardisation of practice, especially protocols, is a major focus in the development of nano- and other advanced materials and their toxicological assessments. A comprehensive evaluation of physical and chemical properties of the material in a realistic media that mimics the natural environment is also an essential requirement.

This output is significant because unlike many other nanomaterials, GO-Au nanohybrids are not yet commercially available, and a standardised protocol for a reproducible well-tested hybrid could support commercialisation. The high yield of the synthesis method further enables using the nanohybrid in ecotoxicological research, which often requires a good quantity of the same material for consistency and reproducibility testing in toxicological studies.

This research paves the way for critical evaluation of the nanotoxicological assessment of GO-Au nanohybrid in biological and environmental systems, especially given that the stability and characteristics of the GO-Au nanohybrid were not impacted by ageing for up to 4 months.

## Figures and Tables

**Table 1:** Analysis of C1s scan by XPS showing the chemical groups in GO.

Functional Groups	Binding Energy (eV)	% Atomic
Aromatic carbon (-C=C-)	284.13	9.98
Aliphatic carbon (-C-C-)	284.96	35.98
Hydroxyl/epoxy (C-O)	286.93	45.68
Ester (COO)	288.67	8.35

**Table 2:** Analysis of C1s scan showing the chemical groups in GO-Au.

Functional Groups	Binding Energy (eV)	% Atomic
Aromatic carbon (-C=C-)	288.93	7.37
Aliphatic carbon (-C-C-)	285.03	35.04
Hydroxyl/epoxy (C-O)	287.09	45.37
Ester (COO)	284.12	9.92
π→π* transition in aromatic	290.67	2.3

## Data Availability

Not applicable.

## Supplementary Materials

The following supporting information can be downloaded at: https://www.mdpi.com/article/10.3390/nano13010033/s1, Figure S1: Line profile of graphene oxide (GO) height obtained from AFM measurements. The red line indicates the thickness of the GO which is 1nm; Figure S2: FTIR Spectra of GO and GO-Au nanohybrid; Figure S3: XPS C1s scan for GO-Au nanohybrid; Figure S4: XPS Survey spectrum for GO-Au nanohybrid; Table S1: Water quality parameters of borehole water media used for dispersion stability experiment; Table S2: Elemental Composition of the Borehole media as measured by ICP-OES; Table S3: Result of the quantification of Au in suspensions of the GO-Au nanohybrid. X, Y and Z are the different batches of GO-Au nanohybrid produced; Table S4: Comparison of the stock concentration and the quantity of AuNPs (concentration and mass) in each of the 3 batches of GO-Au nanohybrid; Table S5: UV-vis measurement of absorbance at 230nm of dispersion stability of GO and GO-Au in ultrapure water, borehole water and high hardness combo media.
