# Supplementary material for: Synthesis and Characterisation of a Graphene Oxide-Gold Nanohybrid for Use as Test Material
Source: Nanomaterials (Basel). 2022 Dec 21;13(1):33. doi: 10.3390/nano13010033 (PMC9824158; doi:10.3390/nano13010033)
Supplement: Supplementary file 1 [file nanomaterials-13-00033-s001.zip › nanomaterials-2024569-supplementary.pdf]

nanomaterials

Supplementary Information

Synthesis and Characterisation of a Graphene Oxide-Gold Nanohybrid for Use as Test Material

Taiwo Hassan Akere [1,2], Aline M. Z. de Medeiros [3,4], Diego Stéfani T. Martinez [3,4], Bashiru Ibrahim [1,2], Hanene Ali-Boucetta [2,*] and Eugenia Valsami-Jones [1,*]

[1] School of Geography, Earth and Environmental Science, College of Life and Environmental Sciences, University of Birmingham, Birmingham B15 2TT, UK; txa880@bham.ac.uk (T.H.A.); bxi824@bham.ac.uk (B.I.)
[2] Nanomedicine, Drug Delivery & Nanotoxicology (NDDN) Lab, School of Pharmacy, College of Medical and Dental Sciences, University of Birmingham, Birmingham B15 2TT, UK
[3] Brazilian Nanotechnology National Laboratory (LNNano), Brazilian Centre for Research in Energy and Materials (CNPEM), Campinas, 13083-100, SP, Brazil; aline.medeiros@lnnano.cnpem.br (A.M.Z.d.M.); diego.martinez@lnnano.cnpem.br (D.S.T.M.)
[4] Centre of Nuclear Energy in Agriculture (CENA), University of São Paulo (USP), Piracicaba, 13416-000, SP, Brazil
* Correspondence: h.aliboucetta@bham.ac.uk (H.A.-B.); e.valsamijones@bham.ac.uk (E.V.-J.)

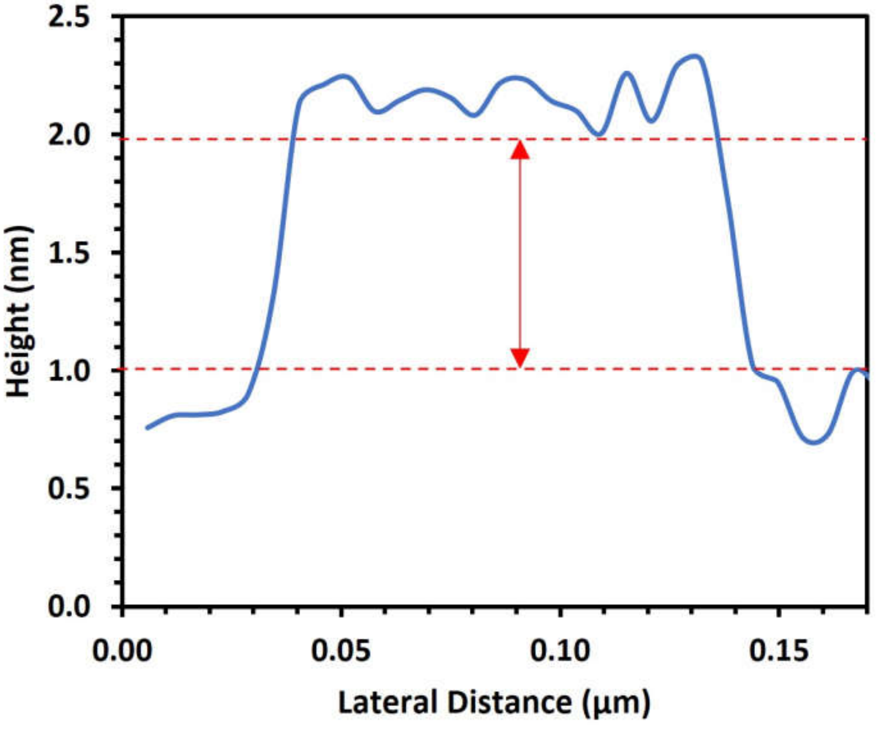

Figure S1. Line profile of graphene oxide (GO) height obtained from AFM measurements. The red arrow indicates the thickness of the GO which is 1nm.

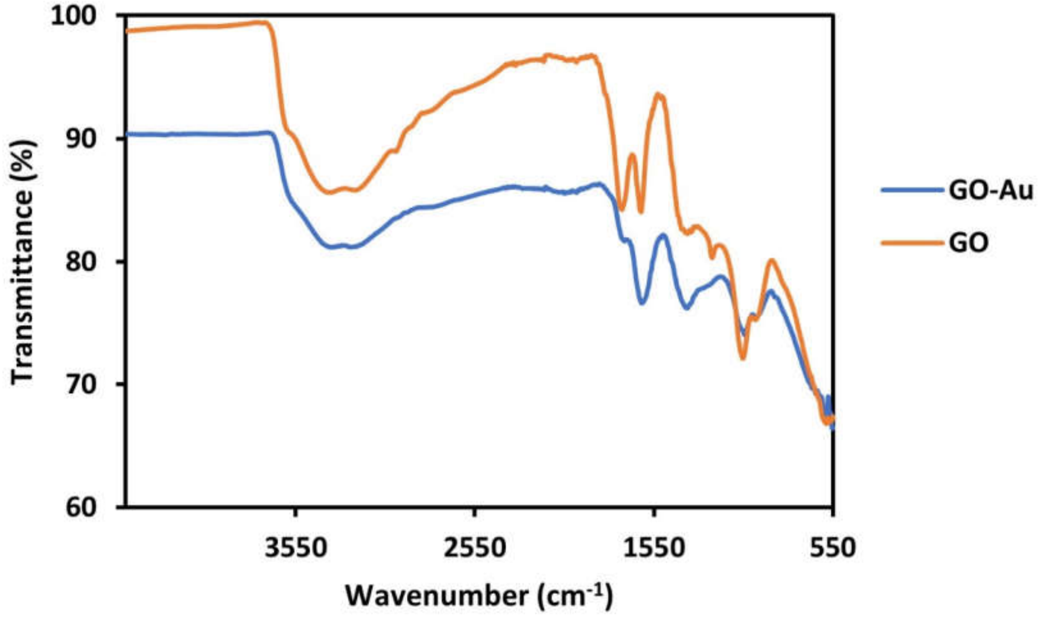

Figure S2. FTIR Spectra of GO and GO-Au nanohybrid.

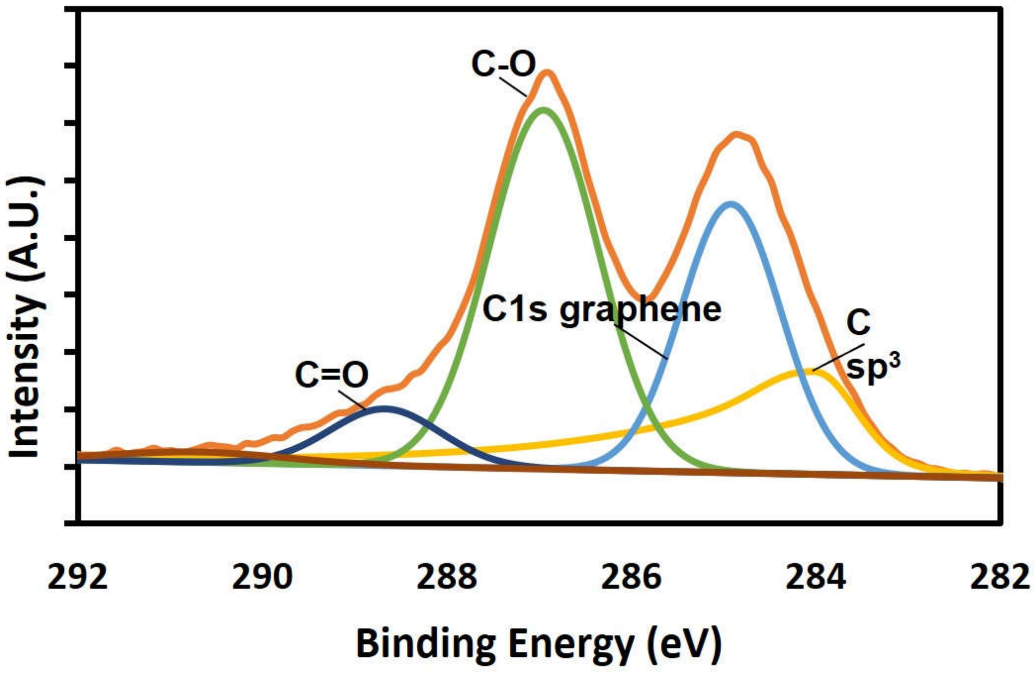

Figure S3. XPS C1s scan for GO-Au nanohybrid.

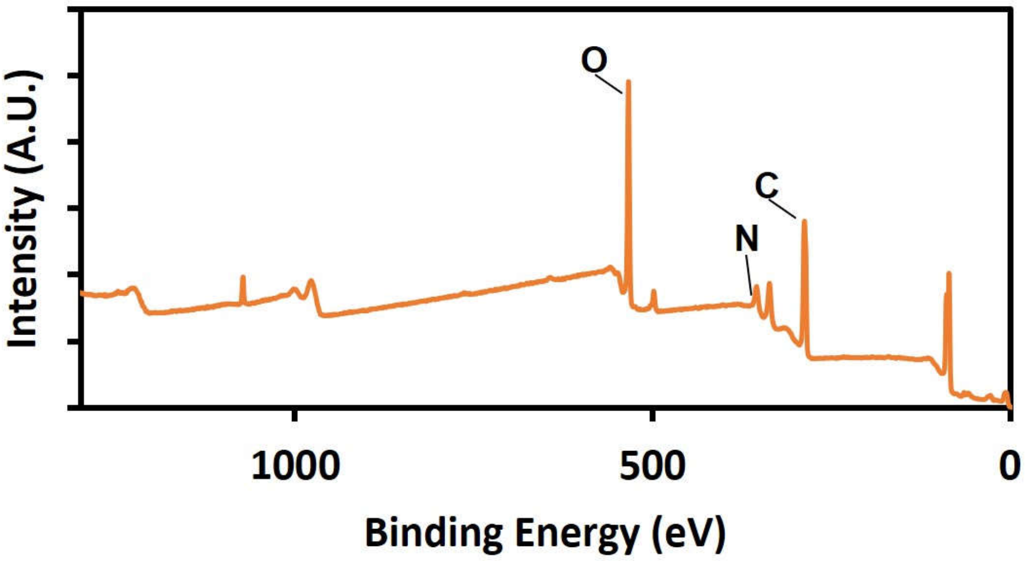

Figure S4. XPS Survey spectrum for GO-Au nanohybrid

Table S1. Water quality parameters of borehole water media used for dispersion stability experiment. Cond = conductivity, DO = dissolved oxygen, Temp = temperature, TOC = total organic carbon, TN = total nitrogen.

Sample ID	pH	Cond (µS/cm)	DO (% Sat)	Temp (°C)	TOC (mg/L)	TN (mg/L)
A	8.03	455.30	108.57	18.43	0.6873	5.9600
B	8.05	449.83	106.50	17.90	0.9493	1.9472
C	8.07	449.93	107.77	18.17	0.8883	6.2777
D	8.06	451.23	105.67	17.57	0.8903	6.0687
E	8.07	451.50	106.33	17.80	0.9884	6.0090

Table S2. Elemental Composition of the Borehole media as measured by ICP-OES.

Sample ID	Ti 48 (ppb)	Mn 55 (ppb)	Fe 57 (ppb)	Ni 60 (ppb)	Cu 63 (ppb)	Zn 66 (ppb)	Cd 111 (ppb)	Hg 202 (ppb)	Pb 208 (ppb)	P 31 (ppb)	Mg 24 (ppm)	K 39 (ppm)	Ca 43 (ppm)
A1	0.6265	0.0343	120.1771	1.5177	0.4768	0.7891	0.0033	3.6525	0.0185	16.8295	17.0929	1.5649	50.6127
A2	-0.2254	0.0198	127.9203	1.1520	0.3512	0.8224	0.0024	1.5368	0.0117	15.8895	18.1787	1.6154	50.3031
A3	2.3615	0.0166	132.9965	1.0800	0.3259	1.0502	0.0009	1.0710	0.0084	15.6777	18.8887	1.6195	49.2164
B1	2.3319	0.0141	140.4039	1.1228	0.3903	1.1968	0.0030	0.8444	0.0083	16.0679	19.6463	1.6338	47.5879
B2	6.3047	0.0141	146.7040	1.1391	0.3446	0.7726	0.0016	0.6727	0.0076	16.1586	20.0361	1.6129	47.4033
B3	5.0909	0.0130	149.6215	1.1844	0.3355	0.9541	0.0024	0.5876	0.0072	16.1498	20.5246	1.6253	48.0946
C1	7.6017	0.0178	149.6959	1.1586	0.3593	1.1833	0.0025	0.5141	0.0068	16.0065	20.6367	1.6158	47.8937
C2	7.4065	0.0184	149.6578	1.1741	0.3550	1.1739	0.0023	0.4675	0.0046	15.7838	20.2727	1.5940	47.1101
C3	8.9336	0.0232	152.2085	1.1732	0.3775	1.2105	0.0016	0.4342	0.0049	15.5422	20.7715	1.6102	47.1611
D1	9.2285	0.0133	155.9447	1.1276	0.3380	0.9162	0.0015	0.3986	0.0045	15.5924	21.1208	1.6349	49.0243
D2	11.9227	0.0152	155.7003	1.1733	0.3169	0.8211	0.0012	0.3682	0.0037	15.7637	21.1289	1.6093	46.7691
D3	11.8417	0.0157	163.4611	1.1991	0.3393	0.8875	0.0008	0.3214	0.0039	15.2921	21.5937	1.6483	47.9672
E1	11.3920	0.0186	149.6093	1.1298	0.4307	1.5395	-0.0006	0.3248	0.0040	14.7385	19.9554	1.5234	44.2731
E2	11.4469	0.0174	154.4847	1.1423	0.3778	1.0758	-0.0008	0.2768	0.0019	14.8148	20.5902	1.5679	45.6040
E3	11.0080	0.0203	157.0267	1.1429	0.3279	0.7723	-0.0002	0.2648	0.0065	15.5459	21.3297	1.6080	47.9792

Table S3. Result of the quantification of Au in suspensions of the GO-Au nanohybrid. X, Y and Z are the different batches of GO-Au nanohybrid produced.

Au Concentration (mg/L)			
Replicates	X	Y	Z
1	0.1319	0.1461	0.1320
2	0.1265	0.1395	0.1236
3	0.1216	0.1447	0.1171
4	0.1276	0.1468	0.1250
5	0.1349	0.145	0.1444
Average	0.128	0.144	0.128
SD	0.005	0.003	0.010
x Dilution factor (10,000)	1,285	1,444	1,284

Table S4. Comparison of the stock concentration and the quantity of AuNPs (concentration and mass) in each of the 3 batches of GO-Au nanohybrid**.**

GO-Au	Stock Conc (mg/L)	Conc of Au in 100µL (mg/l)	Mass of Au in 100µL (mg)
X	2,200	1,285	0.13
Y	4,700	1,444	0.14
Z	3,400	1,284	0.13

Table S5. UV-vis measurement of absorbance at 230nm of dispersion stability of GO and GO-Au in ultrapure water, borehole water and high hardness combo media.

		Ultrapure water (UPW)							
		GO		GO + NOM		GO-Au		GO-Au + NOM	
		Average	SD	Average	SD	Average	SD	Average	SD
230 nm	0 h	1.5461	0.0120	1.6918	0.0062	1.6603	0.0171	1.8504	0.0087
	24 h	1.5807	0.0165	1.7119	0.0407	1.7179	0.1222	1.8401	0.0205
	48 h	1.6922	0.0212	1.8590	0.0292	1.8195	0.0286	2.0010	0.0232

		Borehole Water (BHW)							
		GO		GO + NOM		GO-Au		GO-Au + NOM	
		Average	SD	Average	SD	Average	SD	Average	SD
230 nm	0 h	1.2920	0.0086	1.4377	0.0332	1.6856	0.0167	1.5584	0.0204
	24 h	0.1559	0.0166	0.6500	0.1876	0.1444	0.0092	1.3091	0.1779
	48 h	0.1490	0.0106	0.2622	0.0062	0.1474	0.0143	1.3837	0.0948

		High Hardness Combo (HHC)							
		GO		GO + NOM		GO-Au		GO-Au + NOM	
		Average	SD	Average	SD	Average	SD	Average	SD
230 nm	0 h	1.4479	0.0120	1.6279	0.0559	1.1048	0.0100	1.3771	0.1507
	24 h	1.1102	0.0253	1.2619	0.0593	0.1506	0.0216	1.0210	0.0463
	48 h	1.1278	0.0935	1.2339	0.0461	0.1295	0.0012	0.8664	0.0825
